# Based on voxel-based morphological analysis to investigate the effect of acupuncture-rehabilitation therapy on hippocampal volume and its neuroprotective mechanism in patients with vascular cognitive impairment with type 2 diabetes mellitus: A study protocol

**DOI:** 10.1097/MD.0000000000028187

**Published:** 2021-12-23

**Authors:** Luwen Zhu, Qiang Tang, Li Zhang, Guile Xin, Biying Liang, Yuting Fan, Ying Guan

**Affiliations:** a Graduate School, Heilongjiang University of Chinese Medicine, Harbin, Heilongjiang, China; b Department of Rehabilitation, Second Affiliated Hospital of Heilongjiang University of Chinese Medicine, Harbin, Heilongjiang, China.

**Keywords:** acupuncture-rehabilitation therapy, oxidative stress, type 2 diabetes mellitus, vascular cognitive impairment, voxel-based morphometry

## Abstract

**Background:**

Vascular cognitive impairment (VCI) has a decline in the ability of learning, memory, understanding and execution, which seriously interferes with the daily life of patients. Type 2 diabetes mellitus (T2DM) is an important risk factor for VCI. In recent years, the pathogenesis and treatment of VCI with T2DM have become a research hotspot. Acupuncture-rehabilitation therapy plays an important role in the treatment of various functional disorders of cerebrovascular diseases. In the previous study of the research group, it has been proved from the perspective of imaging that acupuncture-rehabilitation therapy can improve the cognitive function of patients with ischemic stroke. Based on the previous research results, we speculate that acupuncture-rehabilitation therapy not only improves the function of cognitive-related brain regions, but also affects its structure, as well as serum biomarkers highly related to T2DM and cognitive function.

**Methods/Design:**

A partial randomized controlled trial will be conducted at the Second Affiliated Hospital of Heilongjiang University of Chinese Medicine, between July 1, 2021 and December 31, 2023. A total of 80 subjects will participate in the trial, including 25 healthy volunteers, 25 T2DM patients with normal cognitive function and 30 VCI patients with T2DM. They will all have at least 1 functional magnetic resonance imaging scan. Through the calculation and analysis of voxel-based morphological analysis indexes, the changes of hippocampal volume and function in the 3 groups are compared, so as to confirm the correlation between hippocampal structural and functional changes, between T2DM patients and healthy volunteers, as well as T2DM patients with normal cognitive and VCI patients with T2DM. Then the VCI patients with T2DM were randomly divided into 2 groups. The control group was given routine drug treatment to control blood sugar and improve cognitive function, and the experimental group was treated with acupuncture-rehabilitation therapy on the basis of the control group. Patients in both groups will receive serum biomarkers (superoxide organic dismutase, maleic dialdehyde, brain-derived neurotrophic factor) detection and neuropsychological scale (Montreal Cognitive Assessment, Webster Memory Scale IV Chinese version (adult version), functional independent measurement) evaluation before treatment, 4 weeks, 8 weeks, and 12 weeks after treatment. The functional magnetic resonance imaging scan will be performed again after 12 weeks of treatment. To observe the effect of acupuncture-rehabilitation therapy on cognitive function of VCI patients with T2DM.

**Discussion:**

This trial can clarify the effect of acupuncture-rehabilitation therapy on hippocampal volume and its intervention mechanism on oxidative stress injury in VCI patients with T2DM, and clarify the scientific connotation of its neuroprotective mechanism in the VCI patients with T2DM, in order to provide a theoretical basis for the clinical application of acupuncture-rehabilitation therapy in the treatment of VCI.

**Trial registration:**

Chinese Clinical Trial Registry (ChiCTR), ChiCTR2100047803. Registered 26 June, https://www.chictr.org.cn/showproj.aspx?proj=128707

## 1 Introduction

Vascular cognitive impairment (VCI) is a clinical stroke or subclinical vascular brain injury caused by cerebrovascular disease and its risk factors. It involves clinical syndrome with at least one cognitive domain impairment, ranging from mild cognitive impairment to dementia, as well as varying degrees of cognitive impairment caused by mixed pathology such as AD.^[1,2]^ The onset of the disease is hidden, which is mainly manifested by the decline in the ability of learning, memory, understanding and execution, which seriously interferes with the patient's daily life and brings heavy pressure and burden to the patient's family and society. Diabetes mellitus (DM), especially type 2 diabetes mellitus (T2DM) is an independent risk factor for VCI. T2DM can not only cause angiopathy, increase the risk of stroke, but also directly lead to cognitive impairment. Therefore, in recent years, the pathogenesis and treatment of VCI with T2DM have increasingly become a research hotspot.

Cognition is a psychological activity in which individuals know and understand things, including memory, computing power, time-space orientation, executive ability, language understanding, expression and application, among others.^[3]^ Cognitive activities are jointly participated by cerebral cortex, white matter, limbic system, interstitial nuclei, brainstem nuclei, and cerebellum. Different cognitive activities activate different brain regions and subcortical structures. Among them, the hippocampus is most closely related to learning, memory, cognition, and emotion. The left hippocampus plays a dominant role in learning and episodic memory, whereas the right hippocampus is mainly related to non-verbal memory. Therefore, bilateral hippocampus injury will cause severe memory loss. The hippocampus is extremely sensitive to changes in blood glucose and blood oxygen levels. Persistent hyperglycemia and hypoxia will damage the structure and function of the hippocampus and cause abnormal emotional and cognitive behavior and other clinical symptoms.

Diabetes is a group of metabolic diseases characterized by chronic hyperglycemia caused by multiple causes. The mainly clinical manifestations are polydipsia, polyphagia, polyuria, and weight loss. The main pathological manifestations are impaired blood glucose control and insulin resistance.^[4]^ Many studies have shown that T2DM is one of the causes of brain injury, and contributes to vascular cognitive dysfunction particularly. In the study of “Effect of acupuncture-rehabilitation therapy on BOLD-functional magnetic resonance imaging (fMRI) and serum biomarkers in cognitive dysfunction after ischemic stroke patients,” it was found that 19 of 30 nondementia subjects with cognitive impairment after ischemic stroke were complicated with T2DM. It has been proved from the perspective of imaging that acupuncture-rehabilitation therapy can improve the Reho and ALFF values in some brain regions of patients with cognitive impairment after ischemic stroke, strengthen spontaneous functional activities, improve cognitive function.

Based on the above research results, we propose the following scientific questions:

First, acupuncture-rehabilitation therapy can improve the learning and memory function of VCI patients with T2DM, and have a beneficial effect on the volume of hippocampus and the structure of neurons, thus improving cognitive function.

Second, acupuncture-rehabilitation therapy can improve the activity of superoxide orgotein dismutase (SOD) and downregulate the content of malefic dialdehyde (MDA) in peripheral blood of VCI patients with T2DM, and upregulate the expression of brain-derived neurotrophic factor (BDNF), so as to play the role of anti-oxidative stress and brain protection.

Therefore, this study intends to take voxel-based morphological analysis (VBM) as the main analysis method and SOD, MDA, BDNF as the main observation indexes to explore the curative effect of acupuncture-rehabilitation therapy in VCI patients with T2DM from the point of view of imaging and serology.

## 2 Methods/design

### 2.1 Objectives

Taking VBM as the main analysis method and SOD, MDA, BDNF as the main observation indexes, the effects of acupuncture-rehabilitation therapy on hippocampal volume and function of VCI patients with T2DM and its intervention mechanism on oxidative stress injury in peripheral serum were discussed from the perspective of imaging, and the scientific connotation of its neuroprotective mechanism on patients with VCI complicated with T2DM was clarified.

### 2.2 Trial design

**Figure 1:**
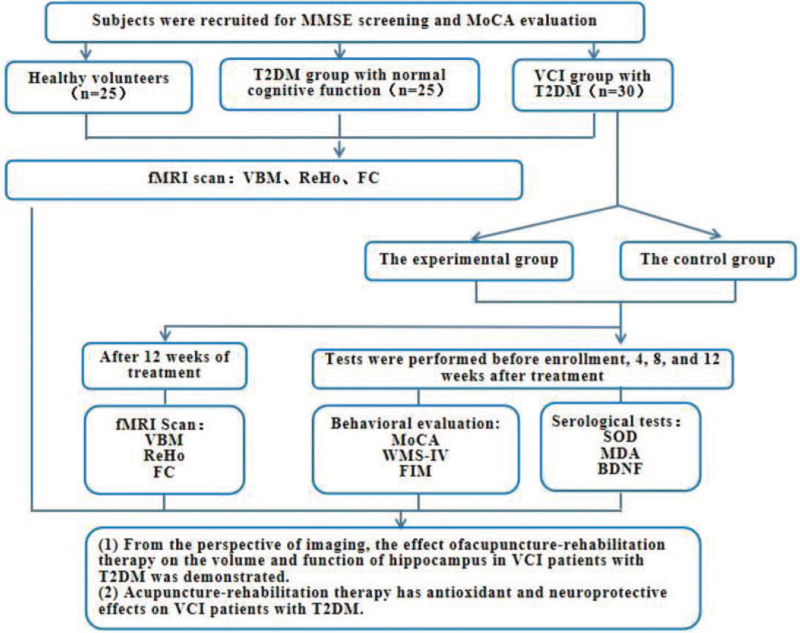
Present the technology roadmap.

This is an outcome assessor- and data analyst-blinded, partial randomized controlled trial conducted at the Second Affiliated Hospital of Heilongjiang University of Chinese Medicine from January 1, 2021 to December 31, 2023. A total of 80 subjects who met the inclusion criteria including 25 healthy volunteers, 25 T2DM patients with normal cognitive function, and 30 patients with VCI combined with T2DM were recruited into the study. First, they were scanned by fMRI, through the calculation and analysis of VBM indexes, the changes in hippocampal volume and function in the 3 groups of patients were compared to determine the difference between type 2 diabetes patients and healthy volunteers, correlation between hippocampal structural and functional changes in patients with normal cognitive T2DM and VCI patients with T2DM. Then, 30 VCI patients with T2DM were randomly divided into the experimental group and the control group, the control group was given routine drug therapy to control blood glucose and improve cognitive function, the experimental group was given acupuncture-rehabilitation therapy on the basis of the control group. The primary outcomes were obtained by VBM calculation and serum biomarkers (SOD, MDA, BDNF) detection, and the secondary outcome indexes were assessed by neuropsychological scales such as Montreal Cognitive Assessment (MoCA), Wechsler Memory Scale IV edition (WMS-IV), FIM, among others. Serum biological indicators and neuropsychological scale will be conducted before treatment, as well as 4 weeks, 8 weeks, and 12 weeks after treatment respectively, fMRI scan will be conducted before treatment and 12 weeks after treatment respectively, to observe the intervention effect of acupuncture-rehabilitation therapy on cognitive function of VCI patients with T2DM. Figure 1 and Table 1*present the technology roadmap and clinical observation flow charts, respectively.*

**Table 1:** Timing of treatment assessments and data collection.

Research stage	Selection period	Baseline period	Treatment period		
Evaluation times	First time		Second time	Third time	Fourth time
Evaluation times point	1 or 0 day before treatment		4 wk after intervention ± 3 days	8 wk after intervention ± 3 days	12 wk after intervention ± 3 days
Inclusion criteria	×				
Exclusion criteria	×				
Informed onsent form		×			
Random grouping		×			
General assessment					
General information		×			
Present medical history/allergic history		×			
History/complications		×			
Physical check		×	×	×	×
CDR		×			
MMSE		×			
IADL		×			
Evaluation of the curative effect index					
Laboratory examination		×	×	×	×
fMRI scan		×			×
MoCA		×	×	×	×
WMS-IV		×	×	×	×
FIM		×	×	×	×
Security assessment					
Life indication		×	×	×	×
Other assessments					
Drug combination		×	×	×	×
Adverse reactions/events			×	×	×
Treatment compliance			×	×	×

CDR = clinical dementia scale, IADL = instrumental activity of Daily living scale, fMRI = functional magnetic resonance imaging, MMSE = Mini Mental State scale, MoCA = Montreal Cognitive Assessment, WMS-IV = Wechsler Memory Scale IV edition

### 2.3 Ethics

This trial was approved by the ethics committee of the Second Affiliated Hospital of Heilongjiang University of Chinese Medicine on 4 April, 2020 (reference number: 2020-K21). All participants will sign an informed consent form, and their real name will not appear in related reports to protect their privacy.

### 2.4 Study setting

All participants in this trial will be recruited from the Second Affiliated Hospital of Heilongjiang University of Chinese Medicine. Interventions for all participants will be carried out in the hospitals where the participants are recruited. The Second Affiliated Hospital of Heilongjiang University of Chinese Medicine will be responsible for this trial coordination and data management.

### 2.5 Sample size

We will conduct this study using a partial randomized controlled trial design, and the main observed outcome will be improvements in VCI patients with T2DM cognitive function. Our previous clinical observation found that 4 weeks of acupuncture-rehabilitation therapy treatment and routine drug therapy can improve scores on the MoCA scale by 3.13 ± 0.75 and 1.53 ± 0.33 points, respectively.^[5]^ Use PASS15.0 software to calculate, with the significance test level set at 0.05 and the test power at 0.9, the required sample size was 13 subjects for each group. Considering a dropout rate of 15%, a total of 30 subjects needed to be enrolled in VCI group with T2DM (15 participants per group). Then the Healthy volunteers group and T2DM group with normal cognitive function group were matched with the corresponding patient volume (25 participants per group). Therefore, a total of 80 subjects were included in this study.

### 2.6 Diagnostic criteria

#### 2.6.1 Diagnostic criteria of T2DM

According to the diagnostic criteria for T2DM in the *Standards of Medical Care in Diabetes-2020*^[6]^ issued by the American Diabetes Association in 2020: FPG ≥126 mg/dL (7.0 mmol/L). Fasting is defined as no caloric intake for at least 8 hours or 2-hour PG ≥200 mg/dL (11.1 mmol/L) during OGTT. The test should be performed as described by the WHO, using a glucose load containing the equivalent of 75 g anhydrous glucose dissolved in water or A_1_C ≥6.5% (48 mmol/mol). The test should be performed in a laboratory using a method that is NGSP certified and standardized to the DCCT assay or in a patient with classic symptoms of hyperglycemia or hyperglycemic crisis, a random plasma glucose ≥200 mg/dL (11.1 mmol/L).

### 2.7 Diagnostic criteria of VCI

According to Guidelines from the Vascular Impairment of Cognition Classification Consensus Study, VCICCS, and Chinese Guide to the Diagnosis and Treatment of Vascular Cognitive Impairment (2019):^[1,2]^

#### 2.7.1 Diagnostic criteria of cognitive impairment

Clinically significant deficits of sufficient severity in at least one cognitive domain (deficits maybe present in multiple domains) and severe disruption to instrumental activity of Daily living scale (IADLs)/ADLs (independent of the motor/sensory sequelae of the vascular event). A clear temporal relationship (within 6 months) between a vascular event and onset of cognitive deficits is only required for a diagnosis of post-stroke dementia.

### 2.8 Diagnostic criteria of vascular etiology

#### 2.8.1 Imaging criteria

Neuroimaging tests need to meet the minimum imaging criteria for the diagnosis of VCI by VASCOG, that is, at least one of the following imaging findings: one large vessel cerebral infarction is sufficient to cause VaMCI, whereas the diagnosis of severe VCI usually requires two or more large vessel cerebral infarct; the presence of an extensive or critical cerebral infarction in the thalamus or basal ganglia region may be sufficient to cause severe VCI; there were >2 luminal infarcts outside the brain stem (The luminal infarction of 1 to 2 key parts or 1 to 2 non-key parts combined with extensive white matter hyperintensity); extensive or fused white matter high signal; cerebral hemorrhage in key areas, or ≥2 cerebral hemorrhage; . a combination of the above forms.

#### 2.8.2 Clinical manifestation standard

Clinical features need to meet one of the following criteria: there is a time correlation between the occurrence of cognitive impairment and ≥1 cerebrovascular events (the occurrence of cognitive impairment should be within 6 months after the cerebrovascular event, and the cognitive impairment is irreversible, and the cognitive impairment is often sudden. With the occurrence of many similar cerebrovascular events, it shows step-by-step progress or volatility, lasting more than 3 months); in the absence of cerebral vascular events, patients should have evidence of significant impairment of information processing speed, complex attention, significant impairment of frontal executive function. The following features can be used as support points: early gait abnormalities, including walking imbalance or repeated falls; early occurrence of frequent urination, urgent urination, or other symptoms that cannot be explained by urinary diseases; personality or emotional changes, such as loss of willpower, depression, or emotional incontinence.

### 2.9 Inclusion criteria

#### 2.9.1 Inclusion criteria of VCI patients with T2DM

1. It conforms to the diagnostic points of T2DM and VCI, and has been confirmed to have suffered from ischemic or hemorrhagic stroke by CT or MRI.
2. With cognitive impairment, the score of the Montreal Cognitive Assessment scale (MoCA-Beijing version) is <26 (if the length of education is <12 years, one point is added to the test results to correct the cultural influence). The overall cognitive function is fully preserved, and the score of Mini Mental State scale (MMSE) ≥24. It did not meet the diagnostic criteria of dementia in the fourth edition of the American Diagnostic and Statistical Manual of Mental Disorders, with a clinical dementia scale (CDR) score of 0.5. Activities of daily living (ADL) were not significantly impaired, and the IADL score ≥16.
3. Can cooperate to complete fMRI scan.
4. The course of stroke ranges from 3 to 12 months.
5. The range of age from 50 to 60 years old, right-handedness.
6. No verbal language dysfunction.
7. Have not received acupuncture or rehabilitation treatment in the past three months.
8. Not accompanied by depression, mental symptoms and infectious diseases.
9. The following categories of drugs were not used within 30 days before screening for cognitive impairment: drugs for the treatment of Alzheimer disease or dementia, anti-Parkinson disease drugs, short-acting antianxiety agents, nerve sedatives or analgesics, antiepileptic drugs, hormones, drugs with significant cholinergic or anticholinergic side effects.

### 2.10 Inclusion criteria for patients with normal cognitive function in T2DM

1. Meet the diagnostic criteria of T2DM, and was confirmed by CT or MRI to exclude stroke.
2. Cognitive function is normal, MoCA score ≥26 points (if the length of education is <12 years, one point is added to the test results to correct the cultural influence). MMSE score ≥24.CDR score is 0.IADL score ≥16.
3. Can cooperate to complete fMRI scan.
4. The range of age from 50 to 60 years’ old, right-handedness.
5. No verbal language dysfunction.
6. Have not received acupuncture or rehabilitation treatment in the past 3 months.
7. Not accompanied by depression, mental symptoms, and infectious diseases.
8. The following categories of drugs were not used within 30 days before screening for cognitive impairment: drugs for the treatment of Alzheimer disease or dementia, anti-Parkinson disease drugs, short-acting antianxiety agents, nerve sedatives or analgesics, antiepileptic drugs, hormones, drugs with significant cholinergic or anticholinergic side effects.

### 2.11 Inclusion criteria of healthy volunteers

1. Do not meet the diagnostic criteria for diabetes, and stroke is excluded as confirmed by CT or MRI.
2. Cognitive function is normal, MoCA score ≥26 points (if the length of education is <12 years, 1 point is added to the test results to correct the cultural influence). MMSE score ≥24.CDR score is 0.IADL score ≥16.
3. Can cooperate to complete fMRI scan.
4. The range of age from 50 to 60 years’ old, right-handedness.
5. Diseases without brain trauma, drug and alcohol dependence that may affect the structure and function of the brain, not accompanied by depression, mental symptoms and other diseases.

### 2.12 Exclusion criteria

1. Subjects with obvious auditory, visual, and language impairments that affect cognitive function test.
2. In the past 3 months, there are acute complications of diabetes such as diabetic ketoacidosis and deep coma, and serious diseases such as heart, lung, liver, kidney, and blood system may affect cognitive function.
3. Subjects with central infection, brain tumor, or other focal injury affecting cognition suffer from Alzheimer disease, Parkinson disease, and other neurological diseases that can cause dementia. A history of severe traumatic brain injury with persistent neurological deficits or known structural abnormalities of the brain.
4. The history of stroke is <3 months or >12 months.
5. Suffered from depression in the past 2 years. the score of self-rating anxiety scale or self-rating depression scale > 50.
6. Have a history of alcohol or drug abuse or dependence in the past 2 years.
7. The following categories of drugs were used within 30 days before screening for cognitive impairment: drugs for the treatment of Alzheimer disease or dementia, anti-Parkinson disease drugs, short-acting antianxiety agents, nerve sedatives or analgesics, antiepileptic drugs, hormones, drugs with significant cholinergic or anticholinergic side effects.
8. Those who were claustrophobic or failed to complete fMRI scans due to other reasons during the course of the test.

### 2.13 Elimination criteria

These will include cases with violation of inclusion or exclusion criteria, patients included in the wrong treatment group, and patients who withdraw from the trials, use prohibited drugs or treatments in combination, and with insufficient data.

### 2.14 Recruitment

All participants in this trial will be recruited from the outpatient and inpatient of the Second Affiliated Hospital of Heilongjiang University of Chinese Medicine, and we also plan to recruit through the hospital website and Wechat official account. During the recruitment process, posters, pictures, and videos related to the study will be produced to help participants understand the purpose of this study, explain the advantages, disadvantages and effects of treatment, and inform participants what they need to do to participate in this study. According to the inclusion/exclusion criteria, we will conduct a preliminary assessment to determine and screen whether the following may be included: participants who are interested in participating in the study, and based on the test results to determine whether they can further participate in the study. Subjects who meet the inclusion criteria will be informed of the details of the trial, and subjects or their legal guardians will be required to sign an informed consent form before treatment. The recruitment of subjects is expected to begin on September 1, 2021 and end in March 2023. It may be completed ahead of schedule if a sufficient number of patients are recruited.

### 2.15 Informed consent

Before the study, the general research process will be explained at the time of participant recruitment. Participants will be informed that participation in the trial is absolutely voluntary and that they can withdraw from the trial at any time. In the case of withdrawal, the collected participant study data will not be deleted and will be used for final analysis. Each participant should obtain and sign written informed consent before receiving any intervention related to this study.

### 2.16 Randomization and allocation

All VCI patients with T2DM will be randomly divided into 2 groups: the experimental group and the control group according to the order of case visit and admission. A specialized statistical analyst who is not a researcher in this study will be entrusted to use PASS15.0 software to generate random-number sequences for randomization. Strips identifying participants by grouping information will be hidden in sealed opaque envelopes with sequential numbers. The envelopes will not be opened until informed consent is obtained. All cases were screened with strict reference to the inclusion and exclusion criteria, and the patients who met the inclusion criteria were randomly selected and grouped by computer until the total number of patients (30) was reached. The cases of healthy volunteers and T2DM patients with normal cognitive function were selected into the group according to the order of medical treatment until the total number of patients (25) was reached. A professional statistician who is not a researcher in the study will produce the distribution order. Participants will be recruited by an independent researcher and assigned to different interventions.

### 2.17 Blinding

Due to the limitations of this protocol and the particularity of acupuncture and cognitive treatment, we were unable to adopt a double-blind study design. However, outcome evaluators and statistical analysts may not know the grouping, so they can be blinded. With the exception of the evaluation process, the outcome evaluators will not contact any participants. In the process of data management and statistical analysis, we will invite independent professionals to handle data management, and the statistical analysis of all data will be performed by professional statisticians who are not the principal researchers. Therefore, this will be a partial randomized controlled trial that blinds the evaluators and statistical analysts of the results.

### 2.18 Intervention

All acupuncture physicians and rehabilitation therapists in this trial have at least 3 years of working experience. Participants in different groups will receive different treatments 6 times a week for 12 weeks. The specific intervention methods are as follows:

Healthy volunteers (HC group): without any intervention.

**T2DM with normal cognitive function group** will be given routine blood glucose control drugs, and anti-platelet aggregation, blood pressure control, and lipid control were given according to patients with different complicated diseases.

**VCI with T2DM group**: the control group was only given routine blood glucose control and cognitive function improvement drug (donepezil 5 mg, once a day, orally), and symptomatic treatment was given according to patients with different complicated diseases. On the basis of the control group, the experimental group was treated with acupuncture-rehabilitation therapy; the specific method is to carry out dual task executive function training was conducted during the period of acupuncture retention at the cluster needling at scalp points.

### 2.19 Cluster needling at scalp points^[7]^

The acupoints were taken from the parietal region, anterior parietal region, and frontal region in the 7-zone division method, the parietal area (from Baihui to Qianding and its 1-inch and 2-inch parallel lines to the left and right respectively), the anterior parietal area (from Qianding to Xinhui and its 1-inch and 2-inch parallel lines to the left and right respectively), and the frontal area (from Shenting to Xinhui, and its 1-inch parallel lines to the left and right, respectively).

**Figure 2:**
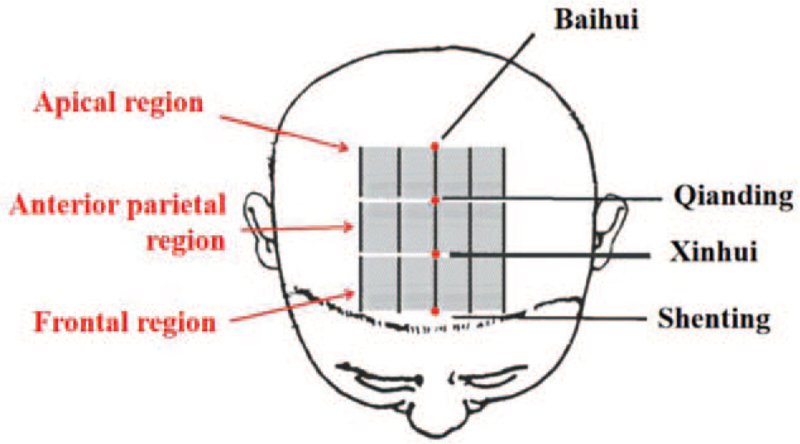
present the Cluster needling at scalp points.

Method of operation: the method of keeping the needle for a long time and sewing intermittently is adopted. After conventional disinfection, the acupoint was punctured from front to back according to the above-mentioned acupoint area, and the acupuncture point was quickly punctured at an angle of 30 degreeto the skin, followed by a slow acupuncture under the cap aponeurosis at an angle of 15 degree to the skin, and about 30 mm was inserted into the needle body. After the needle was twisted, 200 times per minute, each needle was twirled for 1 minute, and the needle was retained for 6 to 8 hours. During the needle retention period, start twirling once every 30 minutes, repeat twice, and then twirl once every 2 hours until the needle comes out. The patients were treated 6 days a week for 12 weeks. Figure 2*presents the Cluster needling at scalp points.*

### 2.20 Dual-task executive function training

In the first task “nailboard test,” the subjects were asked to insert the wooden nails into the holes in the board sequentially with the affected hand within 30 seconds, and the average number of nails inserted three times was recorded. The second task “continuous minus 7,” selects from the data card marked 290–310 randomly, displays the selected initial data at the beginning of the nailboard test, counts backward with 7 as a unit, records with a recording pen, and timing with a timer, carries out a single continuous minus 7 task and a dual task minus 7, respectively, and records the correct number of responses within 30 seconds (all the data correctly minus 7 by the previous data are regarded as a correct calculation). The number of 3 reactions was recorded and the average value was taken. The patients were treated 6 days a week for 12 weeks.

### 2.21 Outcome measures

All participants will be were evaluated for serum biological indicators and neuropsychological scales before treatment, 4 weeks of treatment, 8 weeks of treatment, and 12 weeks after treatment. fMRI scans were performed before treatment and 12 weeks after treatment. All treatment evaluations will be conducted by researchers who will be blinded to the treatment allocation.

### 2.22 Basic characteristic variables

All of the participants’ general status demographic information such as age, sex, educational background, marital status will be attained from baseline questionnaires. Vital signs (pulse, respiration rate, temperature, and blood pressure) will be measured by nurses.

### 2.23 Primary outcome measurement

**VBM:** The VBM is an automatic, comprehensive and objective analysis technique for magnetic resonance imaging (MRI) of brain structures based on voxels. VBM can be noninvasively and accurately study the morphology of living brain and reflect the difference of corresponding anatomical structure by analyzing the changes of each voxel. It is a reliable method to evaluate gray and white matter lesions and plays an important role in the diagnosis of diseases related to cognitive impairment.

**SOD:** The SOD is an active substance derived from living organisms, which can eliminate harmful substances produced by living organisms during metabolism. SOD is mainly used to prevent and treat local cerebral hemorrhage, ulcers, eliminate inflammation, arrhythmia, rheumatism, rheumatoid disease, radiation injury, drug poisoning, and other diseases, and can prevent and treat brain function recognition disorders caused by specific or non-specific brain damage.

**MDA:** The MDA is the final product of lipid peroxide metabolism formed by oxygen free radicals attacking unsaturated fatty acids in biological membranes. It is cytotoxic and can cause cross-linking reactions of DNA, membrane proteins, enzymes, among others, and increase membrane permeability, resulting in changes in the structure, function and metabolism of cell membranes result in damage or death of cells and even the body, which can directly reflect the intensity and rate of lipid peroxidation in the body, and indirectly reflect the content of oxygen free radicals and the degree of nerve cell damage. It is an important biomarker reflecting lipid peroxidation and oxidative stress.

**BDNF:** The BDNF is a protein with neurotrophic effect. It is one of the very important members of neurotrophin family. It was first found in pig brain by Barde et al. Because it can promote the growth, reproduction, regeneration and repair of injured neurons, it mainly has extensive neuroprotective and neurotrophic effects, also is important for neuron survival, maintenance of normal function, and repair after damage. Therefore, it is mainly expressed in the central nervous system, especially in the cortex and hippocampus.

### 2.24 Secondary outcome measures

**MoCA Scale:** The MoCA is presently recognized as one of the cognitive impairment screening scales, which mainly includes attention, executive function, memory, speech, visual space structure, abstract thinking, calculation ability, and other aspects of screening.^[8]^ Compared with the MMSE, MoCA has better sensitivity and specificity for mild cognitive impairment. The total score of MoCA is 30 points. If the subject has been in education for ≤12 years, 1 point is added. A score <26 points is considered to have cognitive impairment.

**WMS-IV:** The Wechsler Memory Scale (WMS) is a set of tests to assess various memory abilities and working memory. It can provide detailed evaluation of clinical memory function for patients suspected of having memory deficits, psychiatric diseases, or developmental disabilities. The WMS was released in 1945 and has been revised to the fourth edition(WMS-Fourth edition) in 2008,^[9]^ WMS-IV has adjusted and improved the structure and content of the original scale, extended the age of the subjects to 16 to 90 years’ old, and added 4 subscales (concise cognitive status assessment, graphic recognition, spatial superposition, symbol breadth), and provide auditory memory, visual memory, visual working memory, instant memory and delayed memory, and total memory quotient to explain the individual's memory ability.^[10]^ Not only to assess memory function, but also to make a brief assessment of cognitive function status.

**FIM:** The FIM is a comprehensive and objective assessment method that reflects the patient's activities of daily living. It selects the most common and useful 18 functional assessment items (including 6 self-care activities, 2 sphincter control, 3 transfers, 2 walking, Communicate 2 items and 3 social cognition items), and determine the appropriate grading scoring method (each item is divided into 7 levels, the highest score is 7 points, the lowest score is 1 point, the total score is the highest 126 points, the lowest 18 points. The higher the score, the better the level of independence, the worse on the contrary.). This scale can comprehensively reflect the patient's function and independent living ability, evaluate and compare the severity of the patient's disability, predict the rehabilitation consequences, formulate rehabilitation goals, select treatment plans, and evaluate the rehabilitation efficacy.

### 2.25 Safety evaluation and adverse events

The safety evaluation mainly focuses on safety indicators and adverse events. The safety indicators will be tested once before and after the treatment, include general physical examination (blood pressure, breathing, pulse, temperature), routine blood tests, routine urine tests, routine stool tests, electrocardiograms, and liver and kidney function tests. Adverse events in this study will be defined as any discomfort, symptoms, or diseases that occurred during this trial, such as fainting, allergies, or pain. If someone faints, researchers will stop all treatments immediately, then keep the patient in a supine position in a ventilated place, and give him/her warm water or sugar water to let him/her fully rest until the body recovers. If allergies or pain occurs, all treatments will also stop immediately, and the patient will be treated symptomatically. All the above detailed information regarding safety evaluations will be reported in detail in case report forms (CRFs), and the impact of all adverse events will be analyzed at the end of the study. Participants who experience adverse events will be compensated accordingly.

### 2.26 Data management

All information of participants will be truthfully, completely, accurately, and timely recorded in CRFs, and codes and initials will be used to replace the information of participants. Special personnel will be asked to manage the relevant data, and each participant's personal information will be kept strictly confidential. Without the explicit permission of the person in charge of the research group, data will not be shared with third parties other than data recorders and data administrators. At the end of the trial, researchers will submit the CRFs on time, and then, the quality control team members will check the completeness and accuracy of the CRFs.

### 2.27 Quality control

To ensure the research quality and data validity, we will conduct unified and standardized training for the study researchers, formulate clinical research SOPs, and ensure that the clinical research consistently follows the operating procedures. Regular monitoring will be used to ensure the study's authenticity and reliability.

### 2.28 Statistical analysis

A third-party medical statistics professional will perform statistical analysis. After the subject research plan and case report form are reviewed by experts, a third party will formulate a statistical analysis plan and determine the statistical software to use.

### 2.29 Specific statistical methods

#### 2.29.1 Statistical analysis of general data, behavioral tests, and blood biomarkers

The metrological data of the measured data were expressed as “mean ± standard deviation,” and the normality test was carried out. If all data were in line with normal distribution (*P* > .05), *χ*^2^ test was used for counting data, *t* test was used for measurement data, double-sample *t* test was used for comparison between the 2 groups, and paired-sample *t* test was used for comparison within the group. The test level was *P* = .05, if *P* < .05 indicated statistically significant difference. The above statistical work is analyzed and processed by specialized statisticians using SPSS25.0 statistical software (SPSS Inc, Chicago, IL).

#### 2.29.2 Statistical processing and result presentation of brain function data

Statistical analysis is carried out by using DPASFA, SPM12, Xjview, Rest, and other software based on MATLAB platform.

The values of VBM, ReHo, and FC of healthy volunteers, T2DM cognitive normal group, and VCI group with T2DM were analyzed by ANOVA, with sex, age and years of education as covariates. The test level was set to: cluster level *P* < .05 (FWE or FDR correction). If *P* < .05 and could at least pass the FDR correction, there was a significant difference between the 2 groups of patients and healthy volunteers, which was statistically significant.

In the VCI group with T2DM, the data of VBM, ReHo, and FC in the experimental group and the control group were compared by double-sample *t* test. Paired-sample *t* test was used for intra-group comparison between the 2 groups before and after treatment. The test level was set as: cluster level *P* < .05 (FWE or FDR correction). If *P* < .05 and could at least pass the FDR correction, it was considered that there was a significant difference between the experimental group and the control group, which was statistically significant.

The statistical results were presented by Xjview and Mricron software.

#### 2.29.3 Related analysis

The significant brain regions detected after treatment in the observation group and the control group will be analyzed by Pearson correlation analysis with the results of neuropsychological scales and serum biomarkers by SPSS software. The statistical significance threshold was set at *P* = .05. Only correlations index of *P* < .05 was considered as valid correlations.

## 3 Discussion

The onset of vascular cognitive impairment is hidden, which is characterized by the decline of learning, memory, visual-spatial, understanding, executive and other abilities decreased in varying degrees after suffering from cerebrovascular disease, which seriously interferes with the patients’ daily life and brings heavy pressure and burden to the patients’ families and society.^[1]^ DM, especially T2DM, is an independent risk factor for VCI. Long-term chronic hyperglycemia can stimulate capillary basement membrane thickening and promote atherosclerosis. In addition, T2DM patients are often complicated with lipid metabolic disorders, high blood viscosity and slow blood flow, which can eventually lead to a decrease in cerebral blood flow, resulting in functional disorders in cognitive-related brain areas such as hippocampus, para-hippocampal gyrus gray, superior temporal gyrus, angular gyrus gray, and so on, so as to accelerate the occurrence of cognitive impairment in T2DM patients.^[11]^ Therefore, T2DM can not only lead to cerebrovascular disease, increase the risk of stroke, but also directly lead to cognitive impairment. Most cross-sectional studies have found that the cognitive function of newly diagnosed diabetic patients began to decline: the MMSE score decreased 0.06 points for every 1mmol/L increase in blood glucose (*P* <.01).^[12]^ For every 1% increase in HbA1C, the standardized Cognitive Assessment Screening Instrument score decreases by 0.06% (95% confidence interval −0.101 to −0.018).^[13]^ Insulin resistance indicates a decline in cognitive ability mainly in the field of memory in patients with prediabetes.^[14]^ Diabetes can also have a synergistic effect with hypertension and heart disease, increasing the risk of cognitive impairment in patients with cerebrovascular disease.^[15]^

Acupuncture-rehabilitation therapy is a new treatment system for stroke dysfunction, which has been studied and summarized continuously by Professor Tang Qiang of Heilongjiang University of Traditional Chinese Medicine after years of experimental and clinical research, and has standardized clinical operation techniques. This method organically combines the cluster needling at scalp points with modern rehabilitation technology to maximize their advantages. On the one hand, the acupuncture effect of the head acupoint prickling can stimulate the cerebral cortex through the skull, regulate the function of the cerebral cortex and reach the focus of responsibility, improve brain function under pathological condition.^[5]^ On the other hand, modern rehabilitation techniques promote the “sprouting” of the damaged brain synapses and the establishment of collateral circulation, so as to compensate the damaged brain tissue around the focus and even the contralateral brain tissue, and give full play to the “plasticity” of the brain. For cognitive dysfunction, acupuncture-rehabilitation therapy means the cluster needling at scalp points and cognitive function training combined with a combination of a treatment.

VBM is an automatic, comprehensive and objective analysis technique for MRI of brain structures based on voxels. The traditional functional magnetic resonance measurement method is to measure and calculate around a pre-selected area of interest, which has the disadvantages of time-consuming, strong subjectivity, poor reproducibility, and inability to perform whole-brain analysis. The VBM can measure and compare the whole brain, directly analyze the original data, without making a priori assumptions, and can quantitatively detect the volume difference of the brain tissue, without subjective influence of the researcher, with automatic, comprehensive and objective and repeatability and other advantages. Some studies have shown that the structural network of patients with T2DM has been significantly damaged at the whole brain and regional level. Mild cognitive impairment patients with T2DM showed more severe impairment of whole brain network efficiency, as well as lower node efficiency and reduced connectivity in multiple regions such as the limbic system, basal ganglia and several cortical structures. In addition, subnetwork damage in patients with T2DM-MCI is characterized by cortical-marginal fibers, as well as commissural fibers and pathways in the frontal, temporal and occipital lobes. The global and node parameters of these changes are significantly related to the cognitive function of patients with T2DM-MCI.^[16]^ Since the slow and hidden decline of cognitive function in patients with T2DM, the detection of blood perfusion at the voxel level of the whole brain will provide sensitive and specific imaging indicators for the early diagnosis and treatment of cognitive impairment, especially for preclinical cognitive impairment. Chen et al^[17]^ found that the volume of temporal lobe, cingulate gyrus and prefrontal cortex in patients with T2DM was significantly smaller than that in normal subjects by VBM analysis. Wu et al^[4]^ found that the volume atrophy of gray matter in different brain regions of T2DM patients with cognitive impairment was higher than that of T2DM patients without cognitive impairment, especially in the middle temporal gyrus and hippocampus. Previous studies also showed that patients with diabetes were positively correlated with the severity of hippocampal atrophy and global brain atrophy (rs = 0.69, *P* < .0001).^[18]^ All the above studies have shown that the changes in the volume of some brain regions are significantly correlated with the cognitive impairment caused by T2DM. However, further studies are needed to confirm whether the occurrence of cognitive impairment is the result of the changes in the volume of a single brain region or the combined effects of the changes in the volume of multiple brain regions.

As mentioned above, oxidative stress plays an important regulatory role in the occurrence and development of diabetes mellitus and the cognitive impairment caused by it. The mechanism may be as follows: activate pancreatic β-cell NFκB signaling pathway, cause cell inflammation, induce cell apoptosis, and inhibit insulin secretion; acts on insulin signaling pathways such as InsR, IRS, PI3K, Akt, GLUT, and induces insulin resistance in liver, muscle, and adipose tissue; the injury causes the accumulation of advanced glycation end products and promotes the occurrence and development of late complications of T2DM.^[19]^

The balance of oxidation and antioxidants in brain plays an important role in maintaining cognitive function. When the level of oxidative stress in the brain is enhanced, excessive oxygen free radicals cause damage to brain cells, resulting in cognitive impairment. Hyperglycemia leading to excessive production of free radicals is the main mechanism of diabetes induced cognitive impairment. Hyperglycemia and the production of free radicals and reactive oxygen species may cause damage to multiple regions of the brain, which may be the pathological basis of cognitive impairment. The main markers of oxidative stress are free radicals. The common indicators of anti-oxidative stress include SOD, MDA, among others.

The BDNF is a growth factor belonging to the neurotrophic family and plays an important role in the nervous system. It was first discovered in pig brain by Yves-Alain Barde and his colleagues. Because it can promote the growth, reproduction, regeneration, and repair of injured neurons, mainly for a wide range of neuroprotective and neurotrophic effects, it is of great significance for the survival of neurons, the maintenance of normal function and the repair after injury, so it is mainly expressed in the central nervous system, especially in the cortex and hippocampus. A meta-analysis showed that patients with cognitive impairment may be associated with a reduction in peripheral BDNF.^[20]^

Therefore, this study intends to use VBM as the main analysis method and SOD, MDA and BDNF as the main observation indexes to explore the effects of acupuncture-rehabilitation therapy on hippocampal volume of VCI patients complicated with T2DM from the perspective of imaging and serology, as well as the intervention mechanism of oxidative stress injury in peripheral blood serum. This study provides a reliable experimental basis for the treatment of VCI combined with T2DM by acupuncture-rehabilitation therapy, and clarifying the scientific connotation of its neuroprotective mechanism, in order to provide a theoretical basis for the clinical application of acupuncture-rehabilitation therapy in the treatment of cognitive impairment.

### 3.1 Dissemination policy

After study completion, the researchers will submit the final data to the National Natural Science Foundation of China in the form of a research report. The findings will be shared with healthcare professionals, the general public, and relevant organizations through the publication of manuscripts and conference presentations.

### 3.2 Trial status

Protocol version number and date: V3.4, 27 March 2020. The recruitment of subjects is expected to begin on September 1, 2021 and completed in March 2023.

## Acknowledgments

We wish to thank all the participants who contributed to this study.

## Author contributions

YG conceived and designed the study, and wrote the manuscript. L-WZ revised the manuscript. QT will direct and supervise the project. G-LX and LZ will investigate the subjects, collect relevant data, recruit and treat patients. LW and Y-TF will sort, analyze and interpret the data. B-YL will apply relevant software to visualize fMRI data. All authors read and approved the final manuscript.

**Conceptualization:** Ying Guan, Qiang Tang.

**Data curation:** Ying Guan.

**Formal analysis:** Ying Guan.

**Investigation:** Ying Guan, Li Zhang, Guile Xin.

**Methodology:** Ying Guan, Luwen Zhu.

**Project administration:** Ying Guan.

**Software:** Biying Liang, Yu Ting Fan.

**Supervision:** Luwen Zhu, Qiang Tang.

**Writing – original draft:** Ying Guan.
